# Reduced Red Blood Cell Count Predicts Poor Survival After Surgery in Patients With Primary Liver Cancer

**DOI:** 10.1097/MD.0000000000000577

**Published:** 2015-02-27

**Authors:** Xiaomeng Xie, Mingjie Yao, Xiangmei Chen, Weiquan Lu, Quanjun Lv, Kaijuan Wang, Ling Zhang, Fengmin Lu

**Affiliations:** From the Department of Epidemiology and Biostatistics (XX, KW, FL), College of Public Health, Zhengzhou University, Zhengzhou; Department of Microbiology and Infectious Disease Center (MY, XC, FL), School of Basic Medical Sciences, Peking University Health Science Center, Beijing; Department of Medical Records (WL), Henan Cancer Hospital; Department of Nutrition and Food Hygiene (QL), College of Public Health, Zhengzhou University; and Department of Hepatobiliary Surgery (LZ), Henan Cancer Hospital, Zhengzhou, China.

## Abstract

Currently, the optimal therapy of primary liver cancer (PLC) remains to be hepatic resection. For better management of the patients, we evaluated the prognostic predicting value of red blood cell (RBC) count, a routine laboratory parameter, on the long-term survival of patients who underwent surgical treatment.

Clinical and laboratory data of 758 patients, who underwent surgical hepatic resection, were retrospectively studied by χ^2^ tests and logistic regression. All patients were enrolled at Henan Cancer Hospital, Zhengzhou, China, from February 2009 to July 2013, and none of them received any other treatments before surgery. Kaplan–Meier survival analysis and Cox proportional hazard models were used to examine the influence of RBC counts on patients’ survival.

The Cox univariate and multivariate analyses showed that preoperative RBC count was an independent risk factor of poor prognosis after surgical treatment. The Kaplan–Meier curves showed that the overall survival (OS) of patients without reduced preoperative RBC counts was significantly better than those patients with reduced preoperative RBC counts (*P* < 0.001). Concordantly, compared with the patients with either reduced preoperative and/or postoperative RBC counts, patients without reduced RBC counts preferred to be low Child–Pugh grades (*P* = 0.0065), which implies a better hepatic function. In addition, low RBC count was found to be significantly associated with patients of female (*P* = 0.003), younger age (*P* =  < 0.001), and with higher AST/ALT ratio (*P* = 0.005).

This study revealed that patients with preoperative RBC counts lower than normal had worse OS rates than those without reduced preoperative RBC counts, perhaps due to the significant correlation of reduced preoperative RBC count to patients’ worse Child–Pugh grade that reflect the loss of liver functions.

## INTRODUCTION

Globally, primary liver cancer (PLC) caused 754,000 deaths in the year 2010, making it the third leading cause of cancer death only after lung cancer and stomach cancer.^1^ More than 80% of annually diagnosed hepatocellular carcinoma cases worldwide occur in sub-Saharan Africa or East Asia. China alone accounts for >50% of the total cases due to endemic hepatitis B virus infection.^2^

Although new therapeutic regimens have developed in recent years, no noticeable improvement of the prognosis of PLC has been archived. The 5-year survival rate after diagnosis was approximately 10%.^3,4^ For those PLC patients who had the applicable profile as defined by the Barcelona Clinic Liver Cancer (BCLC) stage, the optimal therapy remains to be surgical resection.^5–7^ It is generally acknowledged that accurate predicting of a patient's prognosis before surgery is important for the proper management of the patients. A mass of independent prognostic factors, such as serum γ-glutamyl transpeptidase and neutrophil-to-lymphocyte ratio, had been reported as of predicting values for the prognosis of PLC after hepatic resection.^8,9^ Red blood cell (RBC) count is one of the erythrocyte parameters, and a very limited number of studies have reported that it can be used as a screening index or a treatment effect predictor of certain diseases.^10,11^ One report has suggested the relation of elevated red cell distribution width with advanced liver fibrosis in nonalcoholic fatty liver disease.^12^ Unfortunately, no report has connected the alteration of RBC count with human cancers, and the relation between reduced RBC count and poor liver function has never been investigated.

In this study, we evaluated the prognosis predicting value of reduced preoperative RBC count on the long-term survival rate of PLC patients who underwent hepatic resection. In addition, the correlation of reduced RBC counts to patients’ clinical pathological characteristics was also analyzed.

## METHODS

### Study Population and Their Clinical Data Collection

A total of 758 patients who underwent surgical treatment in Henan Cancer Hospital, Zhengzhou, China, from February 2009 to July 2013 were enrolled in this study. All of the enrolled patients were diagnosed with PLC, which was confirmed by pathological diagnosis, and none of them received any chemotherapy or radiotherapy before surgery. This study was approved by the Ethics Committee of Peking University Health Science Center, Beijing, China. Written informed consents were obtained from all the participants. For patients who were children, written informed consents were obtained from their guardians.

### Follow-Up

We followed up the patients by telephoning or visiting the patients or their relatives to consult their medical records. The first follow-up was carried out 1 month after hospital discharge, at the time the patients were advised to return to the hospital for a routine postoperation examination. The second follow-up was at the end of the third month after surgery. After that, the patients were followed-up every 3 months for the first year, then every 6 months for the second and the third year, and once a year for the fourth and the fifth year. If a patient died during the follow-up, we would ask for the exact date of death and cause. The last instance of follow-up was in December 2014. The patients who were alive at the end of the research period were censored.

### Variables

The following data of each patient were collected from the Hospital Episodes Statistics with the permission of the hospital's ethnic committee: sex, age at the surgery, cirrhotic status, presence of tumor thrombi in the portal vein (PVTT), and tumor size. The other laboratory data collected included RBC count, total bilirubin (Tbil) value, alanine transaminase (ALT) value, glutamic oxalacetic transaminase or aspartate transaminase (AST) value, alkaline phosphatase (ALP) value, γ-glutamyl transpeptidase (GGT) value, albumin (Alb) value, albumin/globulin (A/G) ratio, and prothrombin time (PT). Only the data from the last examination before surgery were taken into analysis if multiple laboratory tests were performed. The postoperative RBC count was specified to the examination 1 month after surgery. According to the diagnostic criteria of the hospital, the above biochemistry variables were divided into 3 groups: increased group (higher than the upper limitation of the normal range), normal group (within normal ranges, if in clinical practices there is a range for a laboratory parameter), and decreased group (less than the lowest limitation of the normal range).

In addition, the Child–Pugh grades A to C were determined by the following 5 assessments: bilirubin, Alb, the PT/international normalized ratio, ascites, and hepatic encephalopathy; the BCLC stages were determined by Child–Pugh classification, performance status, and tumor status, and were classified into classes A to D.

### Patient Groups

Among the 758 patients, 724 had accessible preoperative RBC counts. The RBC counts of the patients ranged from 0.38 to 6.91 ×10^12^/L (median 4.2 ×10^12^/L). In clinical practice, the normative values of RBC counts range from 4 to 5.5 ×10^12^/L for male, and 3.5 to 5 ×10^12^/L for female, respectively. Accordingly, the patients were divided into 2 groups based on each patient's preoperative RBC counts: the high preoperative RBC count group (group 1), composed of 551 patients (434 male and 117 female), all of them with normal or even increased preoperative RBC count (RBC ≥ 4 ×10^12^/L for male, or ≥3.5 ×10^12^/L for female), and the low preoperative RBC count group (group 2), composed of 173 patients (150 male and 23 female patients), all with decreased preoperative RBC count (RBC < 4 ×10^12^/L for male, <3.5 ×10^12^/L for female). Of 724 patients, 718 also have available postoperative RBC counts tested at the first returning to the hospital 1 month after surgery. Therefore, the patients were further divided into 4 groups according to each patient's both preoperative and postoperative RBC counts: subgroup 1 with both preoperative and postoperative RBC counts not reduced (n = 348); subgroup 2 with high preoperative RBC count but decreased postoperative RBC count (n = 199); subgroup 3 with low preoperative RBC count but increased to normal or higher postoperative RBC count (n = 48); and subgroup 4 with both low preoperative and postoperative RBC counts (n = 123).

### Statistical Analysis

All variables were expressed as classification variables. The comparison of the variables between the high RBC count group and the low RBC count group was conducted using χ^2^ tests. In addition, a multivariate logistic regression analysis was performed to identify the independent associational variables. χ^2^ tests were also used to compare the relationship between preoperative RBC counts to the Child–Pugh grades. Correlation analysis was carried out to select the analysis variables, in order to avoid multicollinearity. The survival curves of different groups were generated using the Kaplan–Meier method. The differences among the curves were analyzed using the log-rank analysis. The Cox univariate and multivariate analyses were performed to identify the independent factors relevant to patients’ survival. We defined the hazard ratio (HR) as the ratio of mortality between other groups (group 2 and/or 3) and the first group (group 1, control group). The assumptions for calculating the HR have been checked by Wald tests. The analyses were conducted using SPSS 21.0 (Xishu software (Shanghai) company). Differences were significant when the *P* value was <0.05.

## RESULTS

### Identification of the Independent Risk Factors of Poor Prognosis in PLC Patients Who Underwent Surgical Treatment

We collected the preoperative clinicopathologic data and the follow-up information of 758 patients with PLC in Table 1. During follow-up, 112 patients were lost; the remaining 646 patients were successfully followed up and enrolled for the overall survival (OS) analysis. To investigate the risk factors for poor prognosis after surgical treatment, the preoperative RBC count, as well as other 17 potential clinical variables, was analyzed by univariate analysis. As shown in Table 2, preoperative RBC count as well as PVTT, tumor size, Child–Pugh grade, BCLC stage, Tbil value, ALT value, AST value, AST/ALT ratio, ALP value, GGT value, Alb value, A/G ratio, and PT were identified as the candidate risk factors for poor prognosis.

**TABLE 1:**
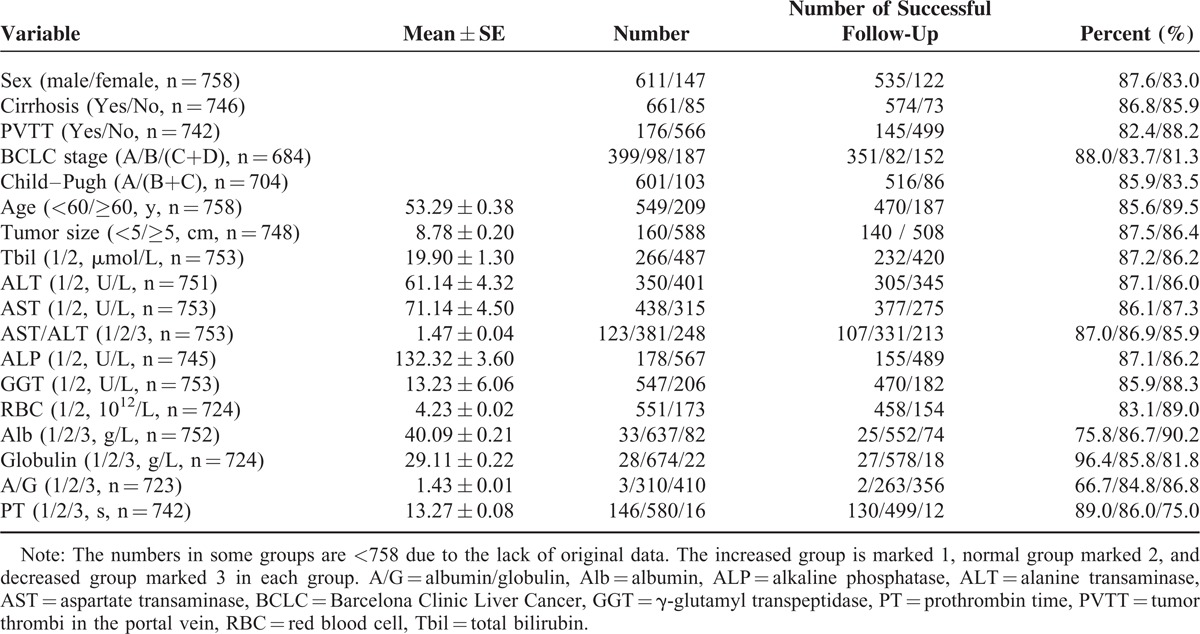
Preoperative Clinicopathologic Data and the Follow-Up Information in Patients With Primary Liver Cancer (n = 758)

**TABLE 2:**
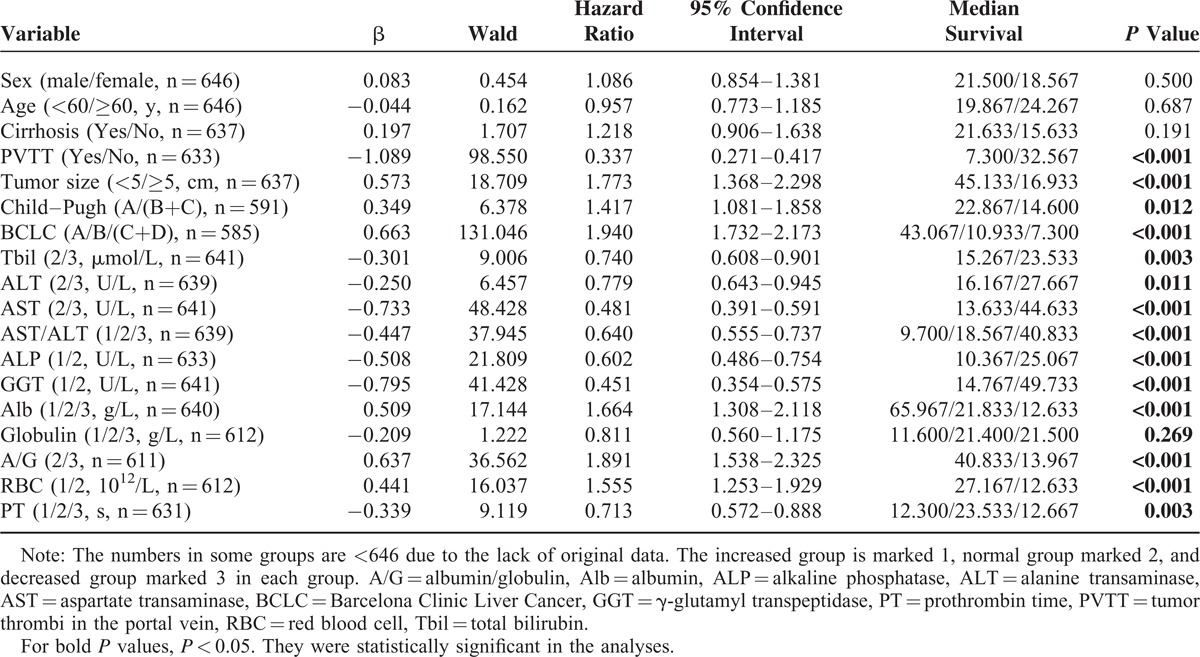
Univariate Cox Proportional Hazard Model for Predictors of Death (n = 646)

Furthermore, through Cox proportional hazards model for multivariable analysis, we identified that decreased preoperative RBC count, advanced BCLC stage, worse Child–Pugh grade, increased AST/ALT ratio, and GGT value were the independent risk factors for poor prognosis in patients with PLC after surgical treatment (Table 3).

**TABLE 3:**
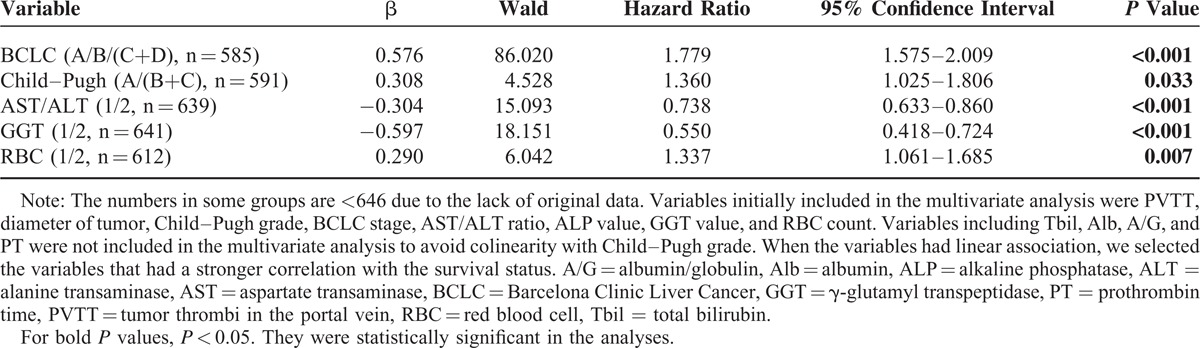
Multivariate Cox Proportional Hazard Model for Predictors of Death (n = 646)

### Lower Preoperative RBC Count Implicated Poor Postoperative Survival

**FIGURE 1:**
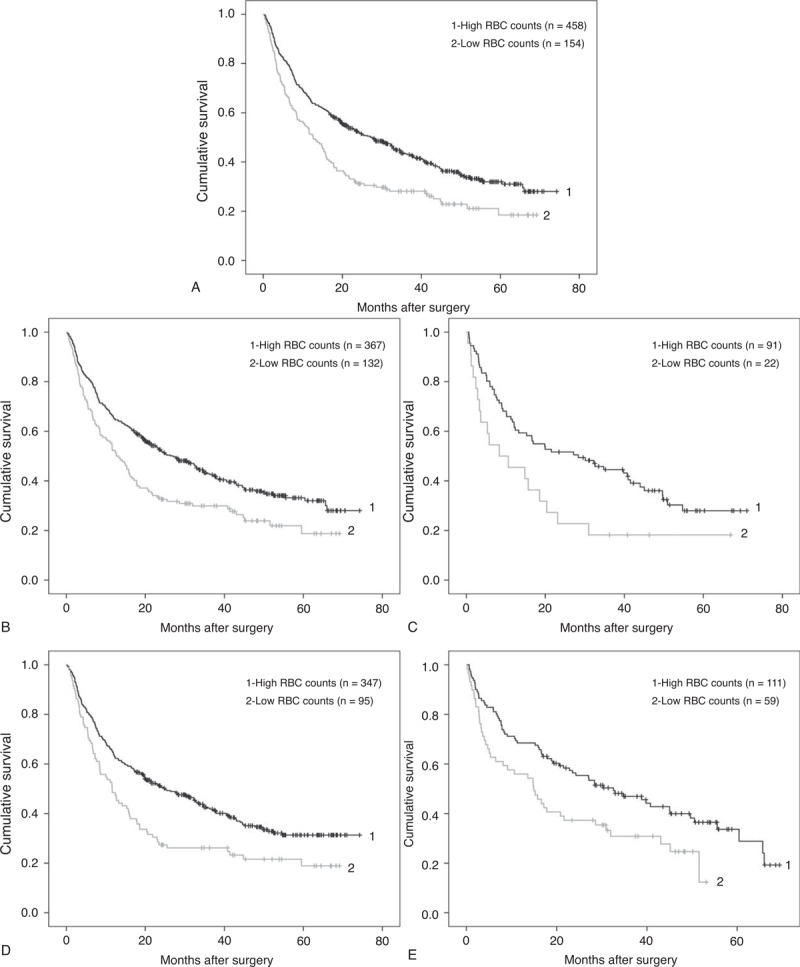
Comparison of overall survival between patients with high RBC counts and patients with low RBC counts. (A) Patients with high RBC counts (group 1, RBC ≥ 4 ×10^12^/L for male, ≥3.5 ×10^12^/L for female, n = 458) and patients with low RBC counts (group 2, RBC < 4 ×10^12^/L for male, <3.5 ×10^12^/L for female, n = 154). Log-rank test: *P* < 0.001. (B) Male patients with high RBC counts (group 1, n = 367) and patients with low RBC counts (group 2, n = 132). Log-rank test: *P* = 0.001. (C) Female patients with high RBC counts (group 1, n = 91) and patients with low RBC counts (group 2, n = 22). Log-rank test: *P* = 0.019. (D) Younger patients with high RBC counts (group 1, n = 347) and patients with low RBC counts (group 2, n = 95). Log-rank test: *P* = 0.001. (E) Older patients with high RBC counts (group 1, n = 111) and patients with low RBC counts (group 2, n = 59). Log-rank test: *P* = 0.010. RBC = red blood cell.

To the best of our knowledge, this is the first study to report that decreased preoperative RBC count is an independent risk factor of poor prognosis in PLC patients who underwent surgical treatment. This discovery promoted us to evaluate its prognostic predicating value of postoperative survival in PLC patients. Of the 724 patients with available preoperative RBC counts, 612 of them were successfully followed up and enrolled for the OS analysis. Kaplan–Meier curves for OS rate of patients with and without decreased preoperative RBC count were plotted in Figure 1A. The OS of the group 2 patients with decreased preoperative RBC counts was significantly poorer than that of the patients in group 1 (HR: 1.374; 95% confidence interval [CI]: 1.092–1.728; *P* *=* 0.007). The 1, 3, and 5-year OS rates of group 2 patients were 31%, 22%, and 19%, respectively. In contrast, the respective OS rates in the group 1 were 52%, 36%, and 26%. In concordance, the median survival time in the group 2 patients was 12.6 months (95% CI: 8.9–16.4), which was significantly <26.6 months (95% CI: 21.2–33.2) of the group 1 patients (*P* < 0.001).

Next, to prove the universal applicability of RBC count as a valuable prognostic parameter, the cohort was adjusted by sex and age. Kaplan–Meier curves analysis conformed that among male patients, the 12.7 months of median OS in patients with decreased preoperative RBC counts was statistically shorter than the 27.1 months of median OS in those without decreased preoperative RBC count (HR: 1.518; 95% CI: 1.198–1.925; *P* *=* 0.001). Similar results were also obtained in female patients; the median OS of patients with and without decreased preoperative RBC counts for females were 8.4 and 28.3 months, respectively (HR: 1.867; 95% CI: 1.097–3.176; *P* *=* 0.019). In China, the mean age of diagnosis with hepatocellular carcinoma was 55 to 59 years.^13^ According to their age at the moment of surgery, the patients were divided into 2 groups using age of 60 year as a cutoff set, while among a subfraction of patients under 60 year old, the median OS of patients with decreased preoperative RBC were 11.6 months, which is statistically shorter than 25.0 months for patients without decreased RBC. (HR: 1.570; 95% CI: 1.205–2.047; *P* = 0.001). Meanwhile, for those patients >60 years old, the tendency remained and the median OS were 15.0 and 32.6 months, respectively (HR: 1.657; 95% CI: 1.123–2.445; *P* = 0.011) (Figure 1B–E). All these data indicated that the OS was significantly poorer in the patients with decreased preoperative RBC counts, as compared with those patients without.

### Impact of Preoperative and Postoperative RBC Counts on the Prognosis of PLC After Surgical Treatment

**FIGURE 2:**
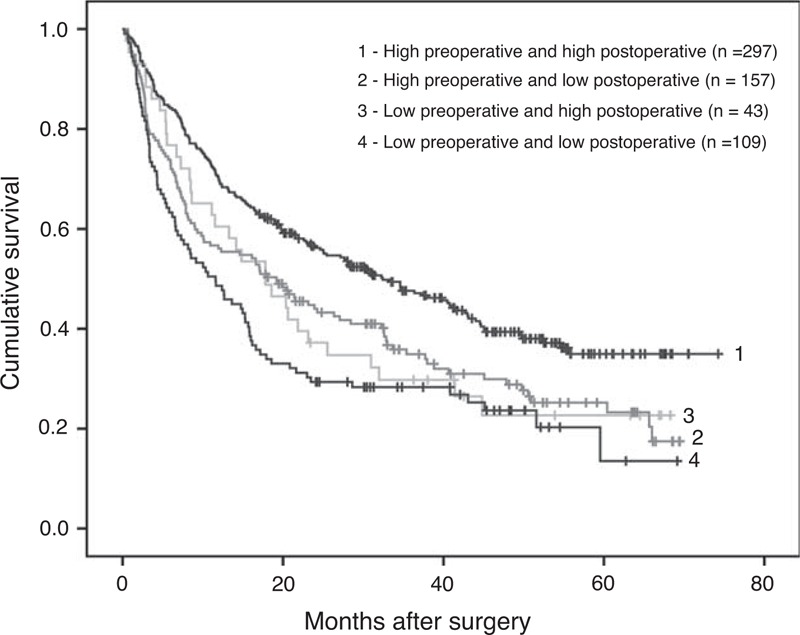
Comparison of overall survival among 4 subgroups (1, 2, 3, 4) of patients. Log-rank test: *P* < 0.001. Subgroup 1: preoperative and postoperative RBC counts were both high (n = 297). Subgroup 2: preoperative RBC count was high but low postoperation (n = 157). Subgroup 3: preoperative RBC count was low but high postoperation (n = 43). Subgroup 4: preoperative and postoperative RBC counts were both low (n = 109).

As preoperative RBC count could potentially be used as a predictor of the OS in PLC patients who underwent surgery, it was worthwhile to take the influence of postoperative RBC count into consideration too. Among the 718 patients with both preoperative and postoperative RBC counts data, 606 of them were successfully followed up. These patients were divided into 4 subgroups according to their preoperative and postoperative levels of RBC counts. Then Kaplan–Meier curve was carried out for OS of each group and the results were plotted in Figure 2. The median OS of the subgroups 1, 2, 3, and 4 were 32.4, 19.2, 17.8, and 11.6 months, respectively. Such differences suggested that patients with both normal preoperative and postoperative RBC counts could expect the best OS, whereas those patients with both decreased preoperative and postoperative RBC counts exhibit the poorest prognosis (*P* < 0.001). However, no statistically significant difference was found among the subgroups 2, 3, and 4.

### Reduced Preoperative RBC Count Was Associated With Worse Child–Pugh Scores and Higher AST/ALT Ratio in PLC Patients

Several previous studies have revealed that certain clinicopathologic variables could impact the OS rate of PLC patients who underwent surgical treatment. Therefore, a variety of clinicopathologic variables were compared between the patients with reduced preoperative RBC count to those without reduced preoperative RBC count. As shown in Table 4, we observed that the following demographic and clinical features were associated with decreased preoperative RBC count according to a univariate analysis: sex, age, cirrhosis, Child–Pugh grade, AST value, AST/ALT ratio, Alb value, A/G ratio, and PT value. In other words, decreased preoperative RBC was more frequently observed in patients who were female, elderly, with hepatic cirrhosis, higher Child–Pugh score, higher AST value and AST/ALT ratio, higher PT value, or lower Alb value and A/G value. We noticed that there was no statistical significance of the association between preoperative RBC count and BCLC grade.

**TABLE 4:**
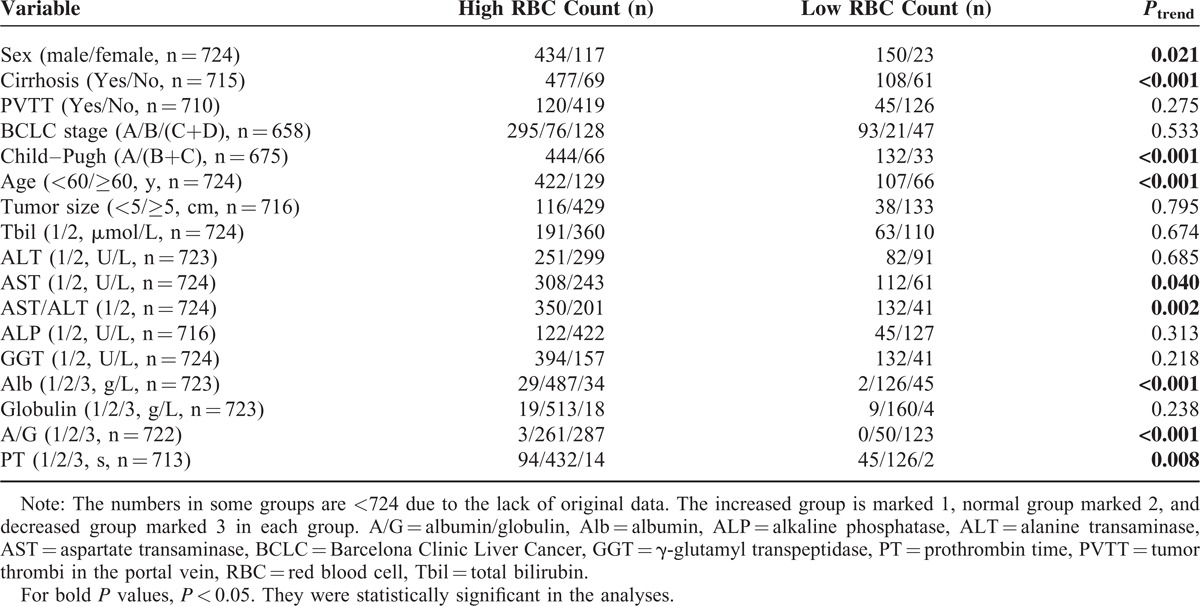
Comparisons of Clinicopathologic Data Between Patients With and Without Decreased RBC (n  =  724)

To validate the associations between the patients’ reduced preoperative RBC count and other demographic and clinical features mentioned earlier, multivariate analysis was performed using logistic regression. As shown in Table 5, the following variables were confirmed as being independently associated with reduced RBC: sex (odds ratio [OR]: 0.447), age (OR: 2.024), Child–Pugh grade (OR: 1.643), and AST/ALT ratio (OR: 0.679). In other words, decreased preoperative RBC was more frequently observed in patients who were female, younger, with a higher Child–Pugh score, or a higher AST/ALT ratio. Although Child–Pugh grade reflects a patient's liver function, as expected, χ^2^ tests revealed an obvious linear trend in the Child–Pugh grade among these 4 subgroups (*P* = 0.0065). In line with this, the proportions of patients scored as Child–Pugh grade A in subgroups 1, 2, 3, and 4 were 88.4%, 83.3%, 71.4%, and 68.8%, respectively.

**TABLE 5:**
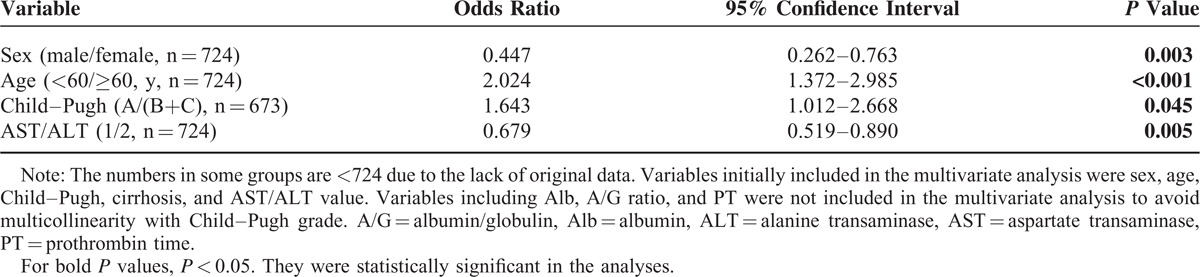
Predictive Variables for Decreased Red Blood Count by Multivariate Analysis Using Logistic Regression Model (n = 724)

## DISCUSSION

In this study, through Cox univariate and multivariate analyses, we identified that decreased preoperative RBC count, presence of portal vein tumor thrombosis, worse Child–Pugh grade, increased AST/ALT ratio, and GGT value were the independent risk factors for poor prognosis in patients with PLC after surgical treatment (Table 3). Some factors among them were in accordance with other studies,^14–17^ which suggested the quality of our current follow-up study cohort. Interestingly, we identified for the first time that reduced preoperative RBC count as an independent risk factor contributed to poor prognosis of PLC patients who underwent surgical treatment. More than that, our data clearly demonstrated that the RBC counts were negatively associated with patients’ Child–Pugh grade, which is a well-known prognostic measure to predict the outcome of PLC patients who underwent surgical resection. Therefore, it was not surprising to find that patients with reduced preoperative RBC counts had shorter survival, just like those with a higher Child–Pugh grade. Moreover, because RBC counts can be examined by routine blood examination, it might be a new and conditional marker to estimate the patients’ general condition and predict the mortality risk of a PLC patent who would undergo surgery.

Because of the difference in the normal RBC counts between male and female, sex was adjusted to observe the OS. As shown in Figure 1B and C, no matter male or female patients, the OS of the patients with decreased preoperative RBC counts was significantly poorer, as compared with that of the patients without decreased preoperative RBC counts. Because the research is about the survival rate after surgery, so age is an indispensable factor, too. Therefore, age of the patients when they underwent surgery was also subjected to adjusting as sex. It demonstrated that no matter among younger or older patients, the OS of the patients with decreased preoperative RBC counts was significantly poorer, as compared with that of the patients without decreased preoperative RBC counts. To further confirm the above observation, the patients were then divided into 4 subgroups with postoperative RBC counts taken into consideration. As expected, a patient with constant normal preoperative and postoperative RBC counts was expected to have a longest survival time after surgery (Figure 2).

It is commonly recognized that certain clinicopathologic variables could impact the OS rate of PLC patients who underwent surgical treatment. At the moment, we cannot provide a mechanistic explanation why a patient's preoperative RBC count linked to his/her postsurgery survival chance. To try to get some clues, we explored the relationship between the reduced preoperative RBC counts with those demographic and clinical features known to be of impact on patients’ postoperative survival rate. As expected, several features were statistically associated with patient's reduced preoperative RBC counts, particularly the patients’ Child–Pugh grade. The significant association between reduced RBC counts with patients’ worse Child–Pugh grades might support the idea that reduced preoperative RBC count might reflect worse liver function. In line with this, we found that there was a strong linear trend of Child–Pugh grades among the 4 subgroups. In addition, AST/ALT ratio, which might be associated with the damage of liver cells, was also associated with reduced preoperative RBC count.

Although it is well known that liver function impacts patients’ survival,^18–20^ it is reasonable to postulate that low levels of preoperative RBC count might reflect poor liver function; it is the latter that attribute to the poor survival of PLC patients. However, the exact mechanisms relevant to the impact between liver functions and RBC counts are not clear. Some studies have suggested that they are interdependent and mutually promotive; therefore, it is possible that they may interplay to prompt the worsening outcome of a patient. Most of the PLC patients accompanied with cirrhosis. As described in 1 report, the hemolytic anemia in cirrhosis may be associated with spherocyte formation, and the mechanism of the hemolysis appears to have consisted in local destruction of red cells, might through their sequestration in the spleen.^21^ Besides, as we know, approximately 25% of the iron required for erythropoiesis was synthesized in the liver in the form of ferritin,^22^ a damage of liver function may lead to the decrease of ferritin, which sequentially impaired the erythropoiesis, primarily in the microcytic anemia.^23^

In addition, we identified that there was no association between RBC count and BCLC stage, and the latter was found to be an independent risk factor for prognosis in patients with PLC after surgical treatment. As we know, BCLC stage system is the preferred tool to evaluate patients with hepatocellular carcinoma and links prognosis assessment with treatment recommendation.^24,25^ However, the prognosis predictive capability of BCLC stage system was limited.^26^ Using RBC count in parallel with BCLC stage system might be helpful to make treatment option recommendations for PLC patients.

We must acknowledge that there are some limitations in this study. For instance, the underlying mechanism attributing to the interdependence between RBC counts and Child–Pugh grade was not solved. We were unable to ascertain if reduced RBC counts worsen liver function, or vice versa. In addition, because we considered a large number of variables in our analysis, it is necessary to make multiple comparisons in a confirmatory study in the future.

In conclusion, this study revealed that reduced preoperative RBC count predicted poor OS rate after surgical resection in patients with PLC. Such prognostic predicting value of preoperative RBC counts was supported by the significant association between decreased preoperative RBC counts and worse liver functions. However, it will be important to validate the prognostic predicting value of preoperative RBC count in the future confirmatory studies.

## ACKNOWLEDGMENT

The authors are very grateful to all the doctors from Tumor Hospital Affiliated to Zhengzhou University for helping them to collect the data.
